# Editorial: Multicellularity in the Cardiovascular System

**DOI:** 10.3389/fcvm.2019.00002

**Published:** 2019-01-23

**Authors:** Paolo Madeddu, Gabor Foldes

**Affiliations:** ^1^Bristol Heart Institute, University of Bristol, Bristol, United Kingdom; ^2^National Heart and Lung Institute, Imperial College London, London, United Kingdom; ^3^Heart and Vascular Center, Semmelweis University, Budapest, Hungary

**Keywords:** multicellularity, microphysiological systems, cell-to-cell interactions, *in vivo* and *in vitro* strategies, cardiomyocyte, endothelial cells

Many cell types exist within the heart and vessels and play key roles in the physiological function of the cardiovascular system. It is unsurprising therefore that successful regeneration of cardiovascular tissues after injury also require the controlled interaction of these cell types. While many functions of non-cardiomyocytes (endothelial cells, smooth muscle cells, pericytes, fibroblasts, and immune cells) in the heart or non-endothelial stromal cells (smooth muscle cells, pericytes, and fibroblasts) in the vessel wall have been clarified *in vivo*, the role of multicellularity in creating physiologically relevant tissues remains mostly unknown. Several factors should be addressed in engineered tissues including humoral signals, cell-to-cell interactions, and matrix materials assessed for their effects on cell survival and enabling cell-to-tissue organization. The purpose of this Research Topic is to provide our readers with the most recent ideas relating to the behavior of these multiple cell types in the cardiovascular system.

There is a growing interest in creating 3-dimensional microphysiological models of the heart and vessels as tool to understand significance of the various cellular and extracellular components. By recapitulating the complex microenvironment that exists in the native tissues, cardiovascular microphysiological systems are proposed as new platforms that could bridge the gap between currently available models and the human body. It is believed that beyond facilitating therapeutic tissue engineering, these systems will enable new insights into tissue morphogenesis, pathogenesis, and drug-induced structural and functional remodeling. Perbellini et al. provide an extensive review of the different components of the heart and their structural, functional, and molecular organization. Zamani et al. report current approaches to create 3D scaffold containing cardiac cells and supporting cells, including bioprinting and organ-on-a-chip echnologies. Tsifaki et al. give a thought-provoking summary on reprogramming approaches toward the cardiovascular regeneration. They also show how to generate a multicellular 3D *ex vivo* model by using these cell products to improve our understanding.

Within this Research Topic, we also collected *in vitro* strategies for physiologically and therapeutically relevant multicellular cardiovascular systems, including stem cell-based approaches. As an example from one of us, Sweeney et al. give an overview how interruption of the interaction between endothelial cells and perivascular cells causes a great variety of genetic and acquired vascular disease. We also gain insight here how novel approaches like derivatives of induced pluripotent stem cells in co-culture can provide potential therapeutic options.

Endothelial cells and cardiomyocytes are the main contributors of the heart multicellularity. The mini-review from Talman and Kivelä describes links between endothelial cells and cardiomyocytes, which uses several means to establish a functional cross-talk instrumental to normal homeostasis and adaptation to stress. Interestingly, the authors report conditions where promotion of angiogenesis are followed by cardiac remodeling. Likewise, cardiomyocyte hypertrophy may result in local ischemia if not accompanied by a parallel increase in surrounding capillaries. The list of factors implicated in this cross-talk comprise cytokines, growth factors, and genetic material. With increasing recognition of cell heterogeneity, it would be relevant to determine if different type of endothelial cells are present in the coronary vascular bed, exerting differential influences according to their topographic (endocardial-epicardial), phenotypic (arterial-venular), expressional, and functional characteristics. This followed by an important paper from Gomez et al. who summarizes cross-talk between cardiomyocytes and immune cells, namely resident and circulating macrophages. Importantly, the article provides a deep insight into the heterogeneity and flexibility of macrophages in response to cardiac injury. This reflects a persisting difficulty in establishing therapies able to block the negative actions of macrophages while preserving the positive actions.

The complex mechanisms of cardiovascular cell-to-cell communications is further showcased by Ontoria-Oviedo et al. They present a significant paracrine crosstalk between cardiomyocyte-derived extracellular vesicles, fibroblasts and endothelial cells under physiological as well as in cardiac remodeling after an ischemic insult. Massaia et al. go further down to individual cell level and explore the advantage of single cell gene expression technologies for studies on cardiovascular development and disease models. The approach unravels heterogeneities, identifies new cell subgroups and helps in organizing cellular hierarchies. The image that emerges from this nice review is different from the views that were widely accepted a few years ago. The most noteworthy change has been the recognition the dynamic nature of molecular interrelationships between cell populations within the heart. This can lead to particularly important findings when investigating spatiotemporal lineage diversification during mammalian development. Along these lines, Malandraki-Miller et al. discuss the importance of metabolic adaptation and the role of substrates in medium during cardiac differentiation process of stem cells and during cell transplantation into the heart. Regenerative cardiology is a large and complex field, we hope that readers of this series of papers in the Frontiers of Cardiovascular Medicine will benefit from several sources of information on current advances and future directions.

## Author Contributions

All authors listed have made a substantial, direct and intellectual contribution to the work, and approved it for publication.

### Conflict of Interest Statement

The authors declare that the research was conducted in the absence of any commercial or financial relationships that could be construed as a potential conflict of interest.

